# P-1887. Improving Vaccine Communication and Decision Making in Pregnancy: A Co-Designed Canadian Intervention

**DOI:** 10.1093/ofid/ofaf695.2056

**Published:** 2026-01-11

**Authors:** Eliana Castillo, Marcia Bruce, Monica Surti, Maria Castrellon Pardo, Medea Myers-Stewart, Andrea Patey, Maoliosa Donald

**Affiliations:** University of Calgary, Calgary, Alberta, Canada; University of Calgary, Calgary, Alberta, Canada; University of Calgary, Calgary, Alberta, Canada; University of Calgary, Calgary, Alberta, Canada; University of Calgary, Calgary, Alberta, Canada; IWK, Halifax, Nova Scotia, Canada; University of Calgary, Calgary, Alberta, Canada

## Abstract

**Background:**

Vaccination in pregnancy (VIP) protects pregnant individuals and their newborns, yet vaccine uptake remains suboptimal. Pregnant individuals face unique decision-making challenges, and communication with their healthcare provider (HCP) is crucial for uptake. We aimed to co-design an intervention to support informed decision-making and vaccine communication in pregnancy.

**Figure 1:**
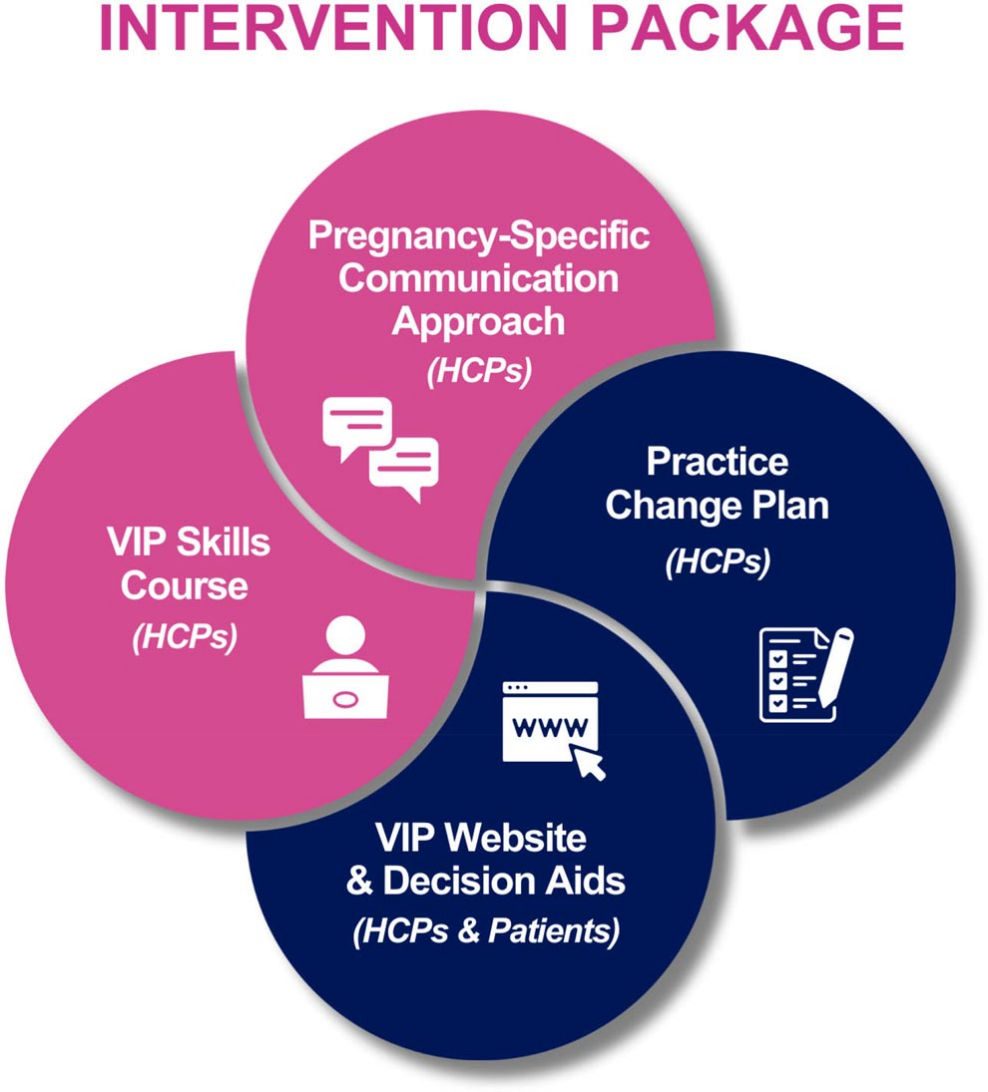
Four components of the Intervention: (1) Pregnancy-specific Communication Approach - helps HCPs deliver a clear vaccine recommendation while supporting shared decision-making (2) VIP Skills Course for HCPs - uses evidence-based behavior change and adult learning techniques to improve HCPs skills and confidence (3) Practice Change Plan - supports HCPs in developing and executing a plan to normalize vaccine communication in their practice (4) VIP Website & Decision Aids - provides evidence-based resources for patients and HCPs

**Methods:**

A multi-method study following the Double Diamond Framework: Discover, Define, Develop, and Deliver. Discover: Partnering with a diverse patient council and a multidisciplinary team of HCPs and intermediaries, we identified barriers and enablers to VIP in Canada through a scoping review, and multi-method inquiry. Define: We defined behavioral change techniques to address barriers and enablers through mapping, rapid review and key informant interviews. Develop: We co-designed and iteratively prototyped intervention components. Deliver: We delivered the intervention following usability and heuristic testing through a feasibility study.

**Results:**

A co-designed intervention that includes four components: (1) pregnancy-specific communication approach to help HCPs deliver a clear vaccine recommendation while supporting shared decision-making (2) VIP skills course for HCPs that uses evidence-based behavior change and adult learning techniques to improve HCPs skills and confidence (3) Practice change plan that supports HCPs in creating a plan to normalize vaccine communication in their practice (4) VIP website with evidence-based resources & decision aids for patients and HCPs. This intervention takes into consideration that Canadian HCPs reported a lack of training and practical tools to improve their vaccine communication skills and that patients reported feeling overwhelmed by vaccine uncertainty. A pre-post feasibility study in one urban outpatient clinic found the VIPC intervention to effectively support HCPs engaging in shared decision-making in a busy practice.

**Conclusion:**

Our innovative co-design approach combined a rigorous problem-understanding inquiry informed by behavioral and implementation sciences with a co-design process grounded on our patient partners and HCPs' perspectives and lived experiences to bridge theoretical frameworks with real world relevance.

**Disclosures:**

Eliana Castillo, MD FRCPC MHSc, Pfizer Canada: Grant/Research Support|Sanofi: Honoraria

